# Development of a test grid using Eye Movement Perimetry for screening glaucomatous visual field defects

**DOI:** 10.1007/s00417-017-3872-x

**Published:** 2017-12-28

**Authors:** N. S. Kadavath Meethal, D. Mazumdar, R. Asokan, M. Panday, J. van der Steen, K. A. Vermeer, H. G. Lemij, R. J. George, J. J. M. Pel

**Affiliations:** 1 Medical and Vision Research Foundation, Chennai, India; 2 Department of Neuroscience, Vestibular and Ocular Motor Research group, PO Box 2040, 3000 CA Rotterdam, The Netherlands; 3 Royal Dutch Visio, Huizen, The Netherlands; 4 Rotterdam Ophthalmic Institute, The Rotterdam Eye Hospital, Rotterdam, The Netherlands; 5 Glaucoma Service, The Rotterdam Eye Hospital, Rotterdam, The Netherlands

**Keywords:** Glaucoma, Screening, Eye Movement Perimetry, Saccadic Reaction Time

## Abstract

**Background:**

Eye Movement Perimetry (EMP) uses Saccadic Eye Movement (SEM) responses for visual field evaluation. Previous studies have demonstrated significant delay in initiation of SEMs among glaucoma patients in comparison with healthy subjects. The aim of the current study was to develop an EMP-based screening grid to identify glaucomatous visual field defects.

**Methods:**

An interactive test consisting of 36 locations and two stimulus contrasts (162 cd/m^2^ and 190 cd/m^2^ on a background of 140 cd/m^2^) was evaluated in 54 healthy subjects and 50 primary glaucoma patients. Each subject was presented a central fixation target combined with the random projection of Goldmann size III peripheral targets. Instructions were given to look at each peripheral target on detection and then re-fixate at the central fixation target while the saccades were assessed using an eye tracker. From each seen peripheral target, the Saccadic Reaction Time (SRT) was calculated for contrast level 162 cd/ m^2^. These values were used to plot Receiver Operating Characteristic (ROC) curves for each test locations and the Area Under the Curve (AUC) values were used to identify the locations with highest susceptibility to glaucomatous damage. Each stimulus location with an AUC less than 0.75 along with its mirrored test location around the horizontal axis were eliminated from the grid.

**Results:**

The mean age was 48.1 ± 16.6 years and 50.0 ± 14.5 years for healthy subjects and glaucoma patients respectively. A significant increase of SRT values by 76.5% (*p* < 0.001) was found in glaucoma patients in comparison with the healthy subjects. From the ROC analysis, ten out of 36 locations meeting the cut-off criteria of AUC were eliminated resulting in a new grid containing 26 test locations. SRT values were significantly different (*p* < 0.05) between the healthy subjects and glaucoma irrespective of the grids used.

**Conclusions:**

The present study resulted in a screening grid consisting of 26 locations predominantly testing nasal, superior and inferior areas of the visual field. An internal validation of the modified grid showed 90.4% of screening accuracy which makes it a potential approach for population based glaucoma screening.

**Electronic supplementary material:**

The online version of this article (10.1007/s00417-017-3872-x) contains supplementary material, which is available to authorized users.

## Introduction

Glaucoma is a progressive optic neuropathy characterized by typical structural alterations of the optic nerve associated with a concomitant visual field defect [1, 2]. Based on a recent estimate, there are 11.2 million people aged 40 years and above with glaucoma in India [3]. Because of its asymptomatic nature, 90% of glaucoma in the community remains undetected [1, 3]. The requirement for additional infrastructure along with financial constraints makes it yet more challenging for developing countries like India to deal with the large number of individuals with glaucoma [3, 4]. Rapid screening for glaucoma in an at-risk population can enable early intervention, thereby reducing visual morbidity and decline in quality of life of an individual with manifest disease [1]. Functional evaluations using perimetry techniques have been considered one such potential approach in glaucoma screening [5].

Standard Automated Perimetry (SAP) is the most accepted diagnostic procedure to quantify glaucomatous visual field defects [2]. In conventional perimetry tests, subjects are required to sustain a steady central fixation throughout the course of testing. This might result in Ganzfeld blank out or Troxler’s fading effect due to neural adaptation [6–11]. This often leads to complaints such as blurred vision, diplopia, inattention, discomfort, hallucination and fatigue. In addition, the fixation requirement contradicts the natural urge of the subject to look at new peripheral stimuli, thus complicating the task. Overall, the approach sets up an unnatural environment for the measurement of human perceptual performance, which can affect the precise measurement of the visual field [6, 8].

Eye Movement Perimetry (EMP) addresses some of these concerns, since it is based on the natural human reflexes. A subject’s performance is based on Saccadic Eye Movements (SEMs), which means the test results include the properties of the oculomotor control system [6, 8]. A key parameter of detected stimuli is the Saccadic Reaction Time (SRT). Past studies have demonstrated reliable and comparable results between SAP and EMP in terms of ability to detect visual field loss [8]. A major benefit of this approach is the elimination of the need for testing for false positive responses [12]. Although a small learning curve exists [13], EMP has potential to become the standard in perimetry for young children and people who have mental or physical limitations to perform conventional perimetry [14, 15].

We have previously shown delayed SRT values in primary glaucoma patients compared to healthy subjects across different eccentricities throughout the tested field of vision [12]. The protocol included all 54 locations tested on the SITA standard 24–2 test pattern of the Humphrey Visual Field Analyzer (HFA). Each point was presented at four different contrasts, thus 216 stimuli in total were shown to each subject [12]. The test duration of the fixed protocol was 12 min per eye, which limited rapid screening for any visual field loss. Therefore, the current study aimed to develop an EMP based rapid and novel screening grid to identify glaucomatous visual field defects in participants with primary glaucoma and age-matched healthy subjects.

## Materials and methods

**Fig. 1:**
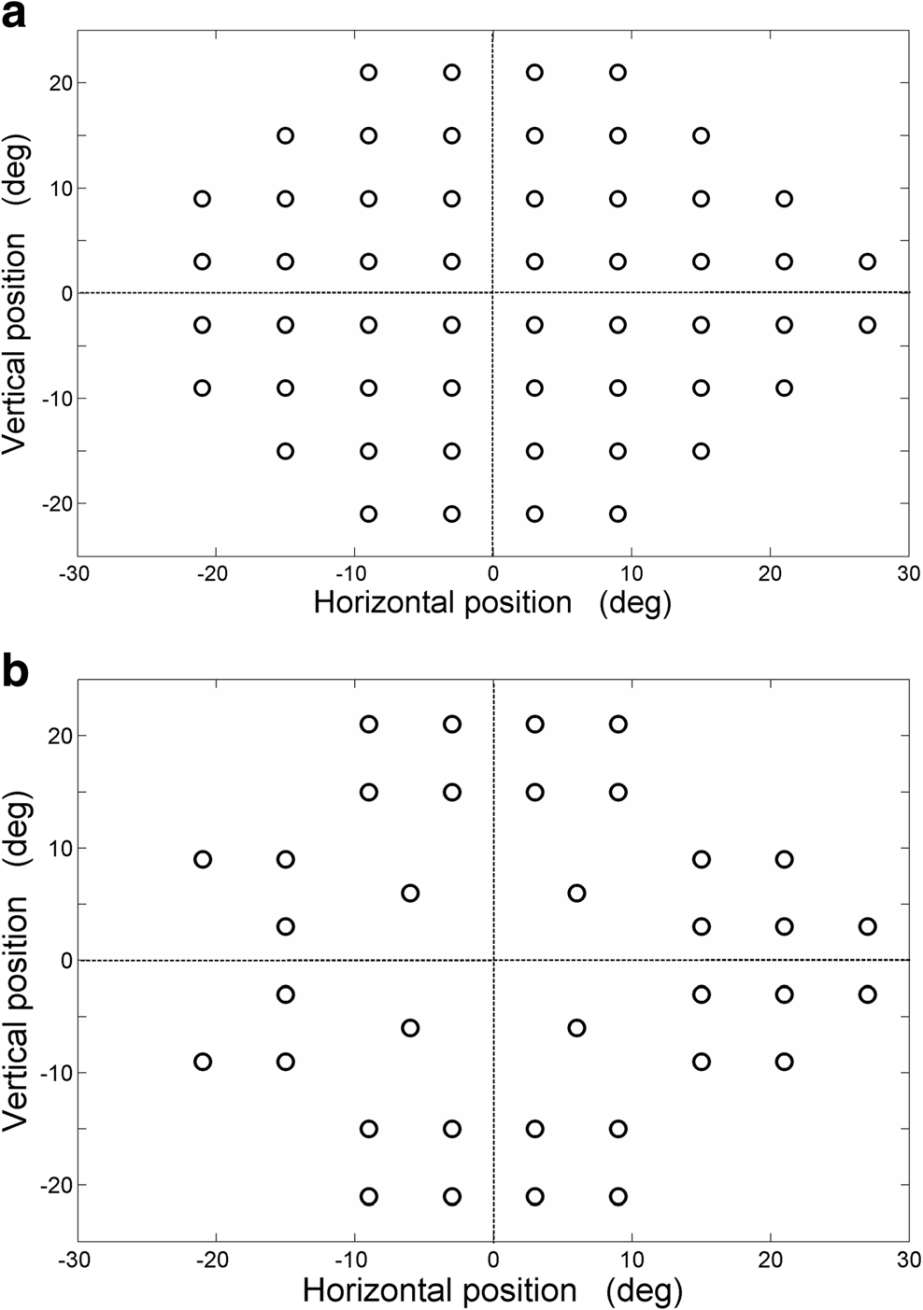
The top panel illustrates the full test grid consisting of 54 locations and the bottom panel illustrates the modified test grid consisting of 36 locations

The full threshold grid (54 locations) used in our previous study (Fig. 1, top panel) followed a fixed stimulus presentation pattern in which each peripheral stimulus appeared for a constant duration of 1.2 s with a 0.2 s time gap between subsequent projections with a total test duration of 12 min [12]. An interactive test version was created wherein each stimulus projection time varied based on the subject’s response. The protocol was programmed in such a way that peripheral stimulus disappeared immediately after the obtainment of an eye movement response from the subject.

The EMP test initially consisted of 4 contrasts varying from subthreshold stimuli (contrast: 192 cd/m^2^) to suprathreshold stimuli (contrast: 276 cd/m^2^) plotted against a 152 cd/m^2^ background. We have previously shown that significant differences in SRTs between healthy subjects and patients with glaucoma were obtained at an intensity level of 214 cd/m^2^. Therefore, we decided to include only 214 cd/m^2^ and 276 cd/m^2^ intensity levels for screening purpose.

### Reduction of test locations

A reduction of the tested locations was obtained in two phases. The first phase was to eliminate irrelevant locations based on the literature and on clinical observation followed by the second phase that focused on an evaluation study in healthy subjects and glaucoma patients using the reduced grid. Based on the evaluation outcomes, another reduction in test locations was obtained by identifying those locations with highest susceptibility to glaucomatous damage. The loss of sensitivity near the foveal region occurs relatively late in the process of glaucomatous damage, when tested with the SAP 24–2 test algorithm [16, 17]. We, therefore, decided to put more emphasis on locations in the peripheral areas of the visual field. The first reduction of the full threshold 54 location test grid (Fig. 1, top panel) was obtained by replacing 16 central stimulus locations (four most central locations in each of the quadrants) with four new test locations. The eccentricity of these four locations was calculated as the mean eccentricity of all 16 locations. In addition, the farthest locations (x = ± 15, y = ± 15) in each quadrant were eliminated along with the two test locations at the blind spot region (15 degree temporal to fixation) [18] thereby achieving an overall 66.7% reduction of tested locations (from 54 locations tested four times to 36 locations tested twice), as illustrated in Fig. 1, bottom panel.

### Evaluation of the 36 grid point

#### Participants

Healthy subjects and patients diagnosed with primary glaucoma aged between 20 to 70 years were recruited at the outpatient department and the glaucoma clinic of Sankara Nethralaya (a tertiary eye care center located in Chennai, India). Healthy subjects were defined as those with an Intra Ocular Pressure (IOP) less than 21 mmHg and a healthy anterior and posterior segment. A dilated posterior segment evaluation was carried out, including assessment of the optic disc by a glaucoma specialist along with visual field evaluation by means of the 24–2 Swedish Interactive Thresholding Algorithm (SITA) standard protocol in the Humphrey Field Analyzer (HFA) to rule out any clinically evident structural or functional signs of glaucoma. Exclusion criteria were a Best Corrected Visual Acuity less than 20/40 for distance and N6 for near, spherical ametropia greater than ±5.00 D and cylindrical ametropia of more than −2.00 D, cataract with a grade more than N2, C1, P1 based on the Lens Opacification Classification System (LOCS) II [19] and any presence of strabismus, nystagmus or retinal diseases [12]. Glaucoma subjects were defined on the basis of the International Society of Geographical and Epidemiologic Ophthalmology (ISGEO) classification [2]. Patients with primary open angle or angle closure glaucoma who had glaucomatous optic disc changes and corresponding reliable and repeatable visual field defects on HFA were included. Reliability criteria were as recommended by the instrument’s algorithm (fixation losses <20%, false positive and false negative errors <33%). Subjects with glaucoma were classified into mild, moderate and severe glaucoma based on their visual field defects by using the Hodapp, Parrish and Anderson’s classification. [16].

All the study participants underwent a comprehensive ophthalmic evaluation that included visual field examination by means of two perimetry techniques. First assessment of each individual’s monocular visual field was done using HFA (model 750; Carl Zeiss Meditec, Dublin) with SITA Standard 24–2 white-on-white protocol followed by our customized and interactive EMP test [Tobii 120, Tobii, Sweden]. Written informed consent was obtained from all the subjects before enrolment. The study adhered to the tenets of declaration of Helsinki and the experimental measures were reviewed and accepted by the Institutional Review Board and Ethics Committee of Vision Research Foundation, Chennai.

#### Measurement setup and procedures

**Fig. 2:**
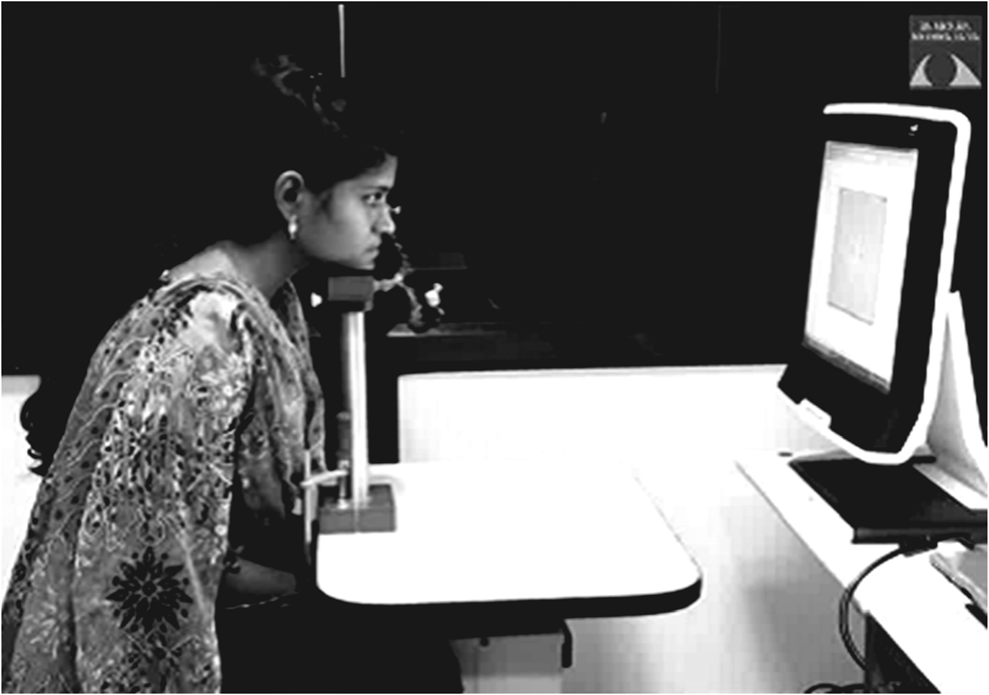
The Eye Movement Perimetry (EMP) test set up consisting of eye tracking device and Thin Film Transistor (TFT) monitor

The EMP setup consisted of a Tobii 120 infrared eye tracking device integrated in a 17 in. Thin Film Transistor (TFT) monitor with a refresh rate of 60 Hz as illustrated in Fig. 2. Each subject was seated in front of the monitor at a fixed working distance of 60 cm. A chin rest was provided to minimize any head movements during the test. A constant testing environment was maintained for all the subjects with dim room illumination and a minimal level of distractions. The right eye was tested first, followed by the left. The non-tested eye was covered with a black polymethyl methacrylate (PMMA) occluder that allowed the infrared eye tracking of the non-tested eye. Customized testing protocol developed for the current study integrated three procedures: a tracking status estimation, a calibration procedure and an interactive EMP test.

Each measurement procedure started with a tracking status estimation that was necessary to ensure appropriate eye alignment and proper gaze tracking. A subject was instructed to look at all the four corners of the screen to ensure negligible eye tracking data loss at each corner. Minor alignment adjustments were done when eye tracking was not optimal. Next, a nine point calibration procedure was done by showing a red circular target to align the subject’s gaze with the presented calibration dots. The calibration procedure was repeated either for preferred locations or for all the nine locations, if poor calibration resulting from various factors such as inaccurate focusing of subjects on the calibration dot, blink artifacts or any hardware/tracking issues, etc. was noted.

After the calibration process, an interactive EMP test was started. A Software Development Kit (SDK) was used to assess real time gaze positions at a 60 Hz sample rate. The test started with plotting a Goldmann size IV central fixation target (green color) on the eye tracker’s monitor with background illumination of 152 cd/m^2^. Meanwhile, real-time gaze tracking was used to monitor correct central target fixation. A peripheral stimulus was projected after a consistent central target fixation for at least 0.5 s. Goldmann size III peripheral stimuli of intensity 214 cd/m^2^ and 276 cd/m^2^ appeared sequentially by using an overlap paradigm, which meant that the central fixation target was kept visible to the subject. A peripheral stimulus was plotted for a maximum duration of 1.2 s. Yet, this stimulus was removed when it was fixated within 1.2 s. All subjects were encouraged to look at detected peripheral targets followed by refixating at the central fixation stimulus. To test the visual field up to visual angles of 27 degrees horizontally and 21 degrees vertically, the central fixation target was repositioned during the test to four eccentric locations. During the presentation of peripheral stimuli, gaze data was collected and stored for analysis.

#### Data analysis and statistics

**Fig. 3:**
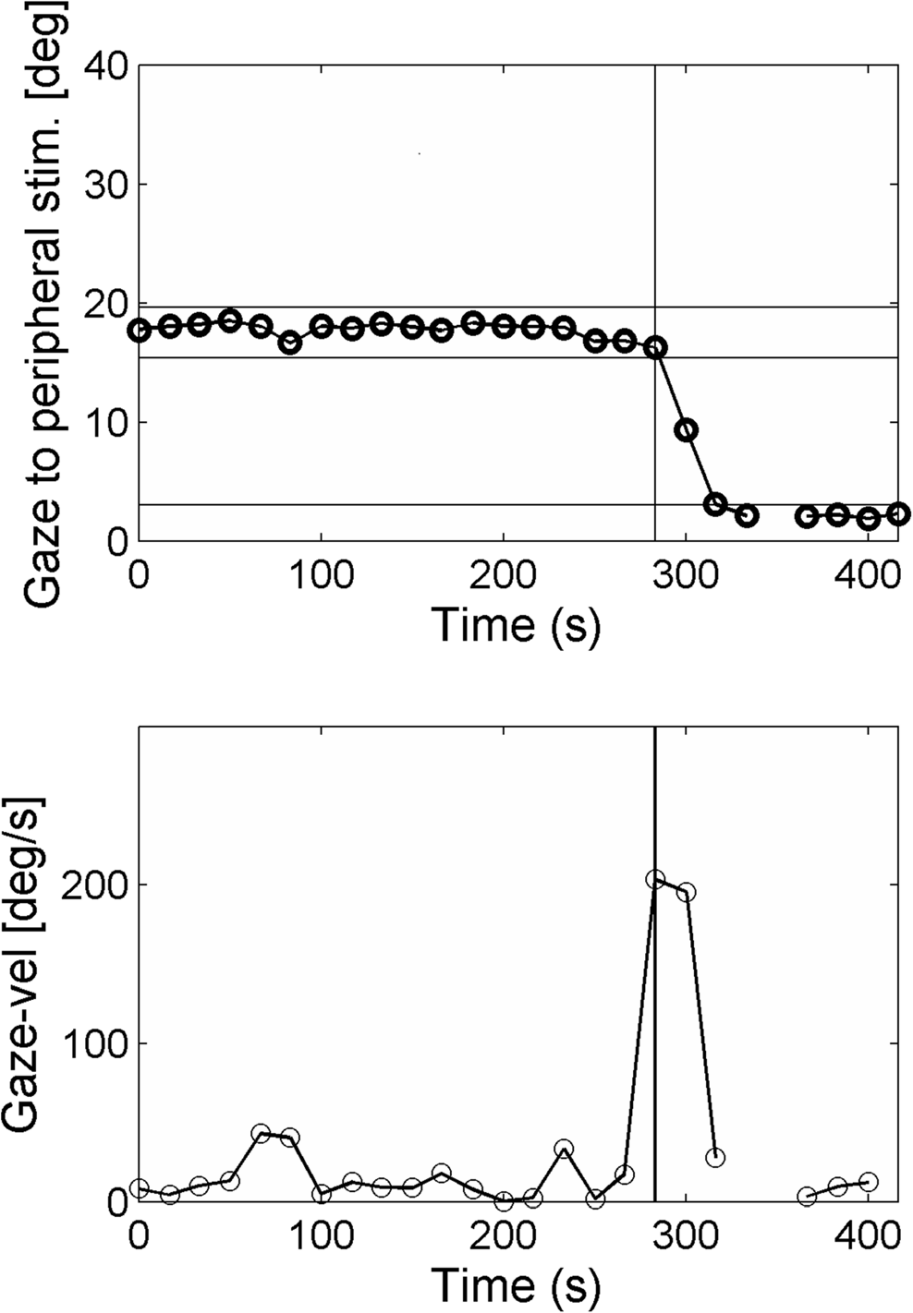
Illustration of the Matlab analysis window used for analyzing the trajectory and time course of a saccade aimed at a peripheral stimulus projected at location x = 3, y = 15

A custom-written Matlab program was used to analyze and calculate the properties of SEMs. Figure 3 illustrates the trajectory and time course of a saccade aimed at a peripheral stimulus projected at location x = 3, y = 15. At the moment of presenting this stimulus, the gaze was on the central fixation target (x = 0,y = 0). A peripheral stimulus obtained was labeled as “seen” if the following criteria were satisfied: (1) fixation of the central target was followed by a peripheral stimulus fixation; (2) the angular disparity between the direction of the primary SEM and the peripheral stimulus location was less than 45 degrees; (3) the amplitude of the primary SEM covered more than 50% of the total target distance. A peripheral stimulus was classified as “unseen” when: (1) fixation remained on the central fixation target; (2) the angular disparity between the direction of the primary SEM and the peripheral stimulus location was larger than 45 degrees (indicating search behavior). A peripheral stimulus was classified as “invalid” when gaze data was poor due to blinking or pupil tracking failure. For the events labeled as “seen”, SRT was defined as the time between the onset of the target stimulus and the initiation of the SEM (Fig. 3). This was determined based on a velocity criterion by calculating the time at which the eye velocity cross 50 degrees per second [13, 20].

Statistical analyses were performed by using SPSS (Statistical Package for Social Sciences, version 15, Chicago, IL, USA). The right eye was considered for analysis. Tests for normality were carried out for all the continuous variables and appropriate parametric/non parametric tests were utilized. Type 1 error was kept at 5% level and all the tests used were two-tailed. Logarithmic transformation of SRT values in milliseconds was done to reduce the skewness of the data, thereby achieving a normal data distribution. An independent T-test was used to compare SRTs between healthy subjects and glaucoma patients. The pattern of eye movement responses was compared between healthy subjects and glaucoma patients by calculating the percentage of reliably seen, unseen and invalid subject responses. The stimulus locations were divided into central and peripheral zones comprising of four locations and 32 locations respectively. All the 36 tested locations were represented by their “x” and “y” coordinates (Supplementary figure 1). Of each location, the obtained SRTs by projecting 214 cd/m^2^ stimuli were used to construct Receiver Operating Characteristic (ROC) curves. The Area Under the Curve (AUC) obtained for 32 test locations in the peripheral zone were considered as an index of accuracy in classifying diseased and non-diseased individuals. The AUC values obtained for each location were inspected for determining the minimum AUC cut-off value below which the points were eliminated along with their mirrored test locations around the horizontal axis for the maintenance of horizontal symmetry.

After designing the reduced grid, SRT values were obtained from both 36 location grid and the modified 26 location grid. Differences in mean SRT values between healthy subjects and glaucoma patients were analyzed using an independent t-test. One-way analysis of variance (ANOVA) was done to compare the mean SRTs across the four diagnostic categories (healthy, mild, moderate and severe) using the SRT values obtained from both grids.

Some healthy subjects had increased SRT values and some glaucoma patients had SRT values that fell within the normal range. A fivefold cross validation technique was adapted to estimate the classification accuracy as a means of internal validation of the modified grid. The analysis began with generating five (k = 5) equal sized random subsets from the original data set. Of the five samples, a single subsample was retained as the validation data for the testing model, and the remaining four subsamples (k-1) were used as training data. The cross validation process was then repeated five times, with each of the five subsamples used exactly once as the validation data thereby calculating five SRT cut-off values using binary logistics (SPSS). These cut-off values were averaged to compute a single estimation of classification accuracy. Finally, to investigate the test duration of the modified grid, another five healthy subjects and 5 glaucoma patients underwent the modified EMP screening test.

## Results

A total of 104 participants were recruited that included 54 age-matched healthy subjects and 50 glaucoma patients. The demographic details of the subjects are presented in Table 1.

**Table 1:** Demographics of the study population

Subject characteristics	Healthy subjects (*n* = 54)	Patients (*n* = 50)
Mean age (SD) in years	48.1 (16.6)	50.0 (14.5)
Gender (in percentage)		
Male	31 (57.4%)	38 (76.0%)
Female	23 (42.6%)	12 (24.0%)
Mean Intraocular Pressure in mm Hg (SD)	14.25 (2.58)	16.15 (3.69)
Mean Cup-to-Disc ratio (SD)	0.39 (0.13)	0.66 (0.17)

The glaucoma patients consisted of 24 (48%), 17 (34%) and 9 (18%) individuals with mild, moderate and severe forms of the disease, respectively. In Table 2, the test performance is summarized by analyzing the percentage of seen points, unseen points due to visual field defects and invalid points due to poor gaze tracking.

**Table 2:** Pattern of eye movement response for healthy subjects and glaucoma patients in both the stimulus contrast levels

Eye movement response	Seen		Unseen		Invalid	
	162 cd/m^2^	190 cd/m^2^	162 cd/m^2^	190 cd/m^2^	162 cd/m^2^	190 cd/m^2^
Percentage of response from healthy subjects (n = 54)	90.5	97.7	6.9	1.4	2.6	0.9
Percentage of response from glaucoma patients (n = 50)	57.3	68.6	30.8	22.2	11.9	9.2

**Fig. 4:**
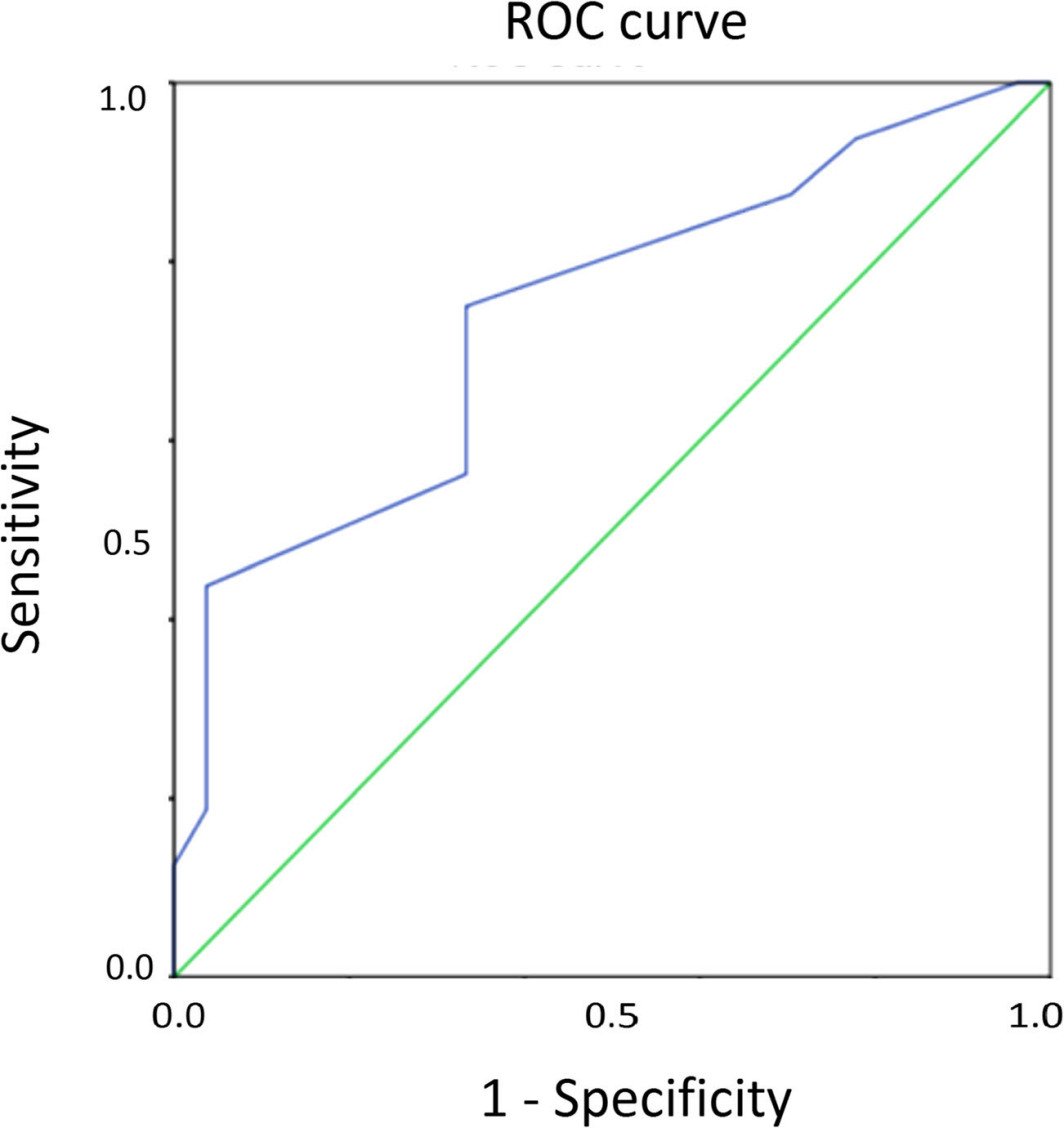
Receiver Operating Characteristic (ROC) curve obtained for location x=21,y=9 with an Area Under the Curve (AUC) of 0.73

**Fig. 5:**
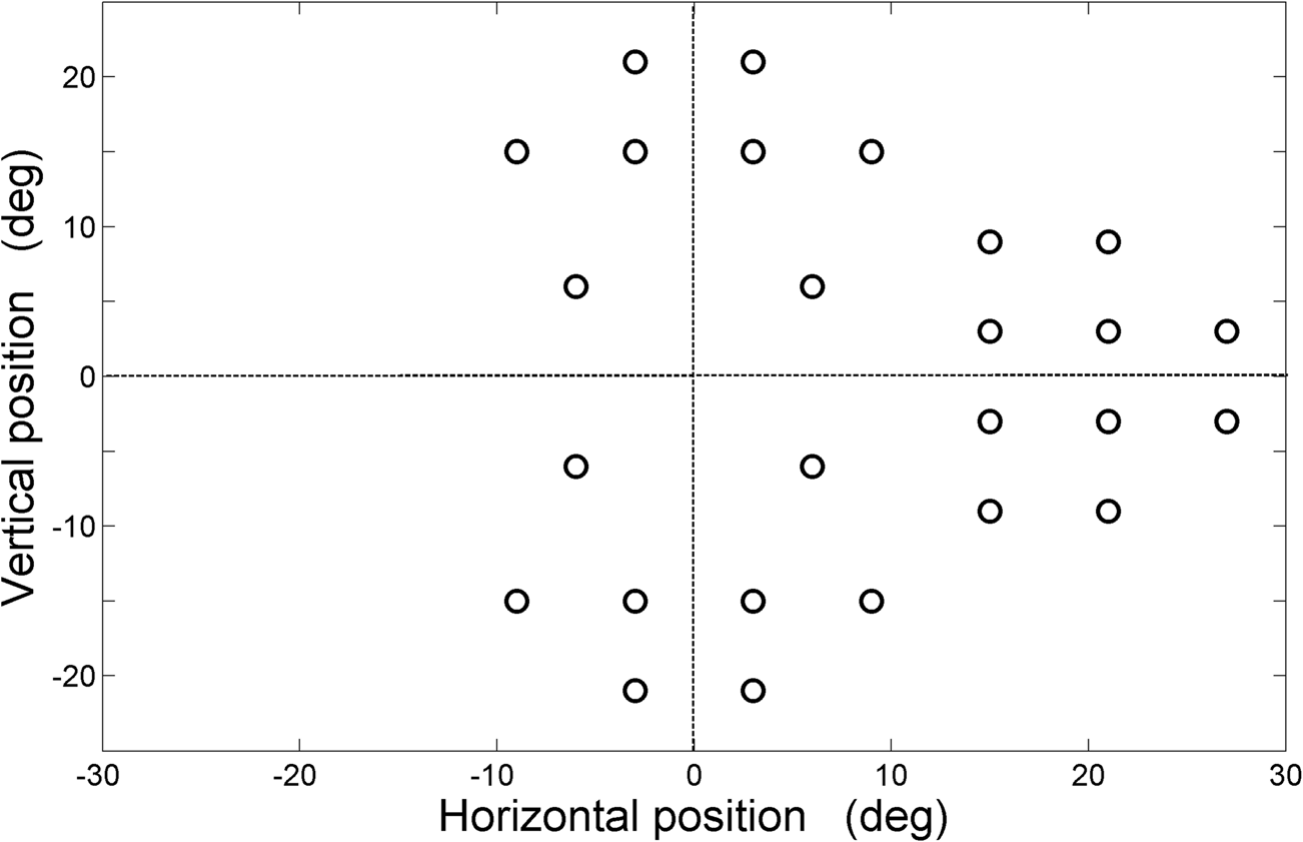
New stimulus grid for the left eye with 26 test locations

Supplementary table 1 describes the AUC calculated for each stimulus locations in the central and peripheral zones. An illustration of one of the point wise ROC analysis outputs is presented in Fig. 4. The AUC values calculated for four central stimulus locations (x = +/−6 and y = +/−6) ranged from 0.71 to 0.81. By inspecting the range of AUC values obtained for the peripheral zone, a cut-off value of 0.75 was decided. We found ten peripheral locations with an AUC below this cut-off and these locations were eliminated from the test grid, resulting in a 26 location grid as in Fig. 5 (illustrates new stimulus grid for the left eye with 26 test locations).

### Comparison of SRT values obtained from the 36 and 26 location grid

The mean SRT values obtained in the 36 location grid were 427 ± 113 ms and 754 ± 194 ms among healthy subjects and glaucoma patients, respectively which was statistically significantly different (*p* < 0.001; Independent t-test). In the 26 location grid, the mean SRT values were 435 ± 116 ms for healthy subjects and 806 ± 234 ms for glaucoma patients. Again, this difference was statistically significantly different (p < 0.001; Independent t-test). SRT values obtained from both the grids did not show any significant difference.

**Fig. 6:**
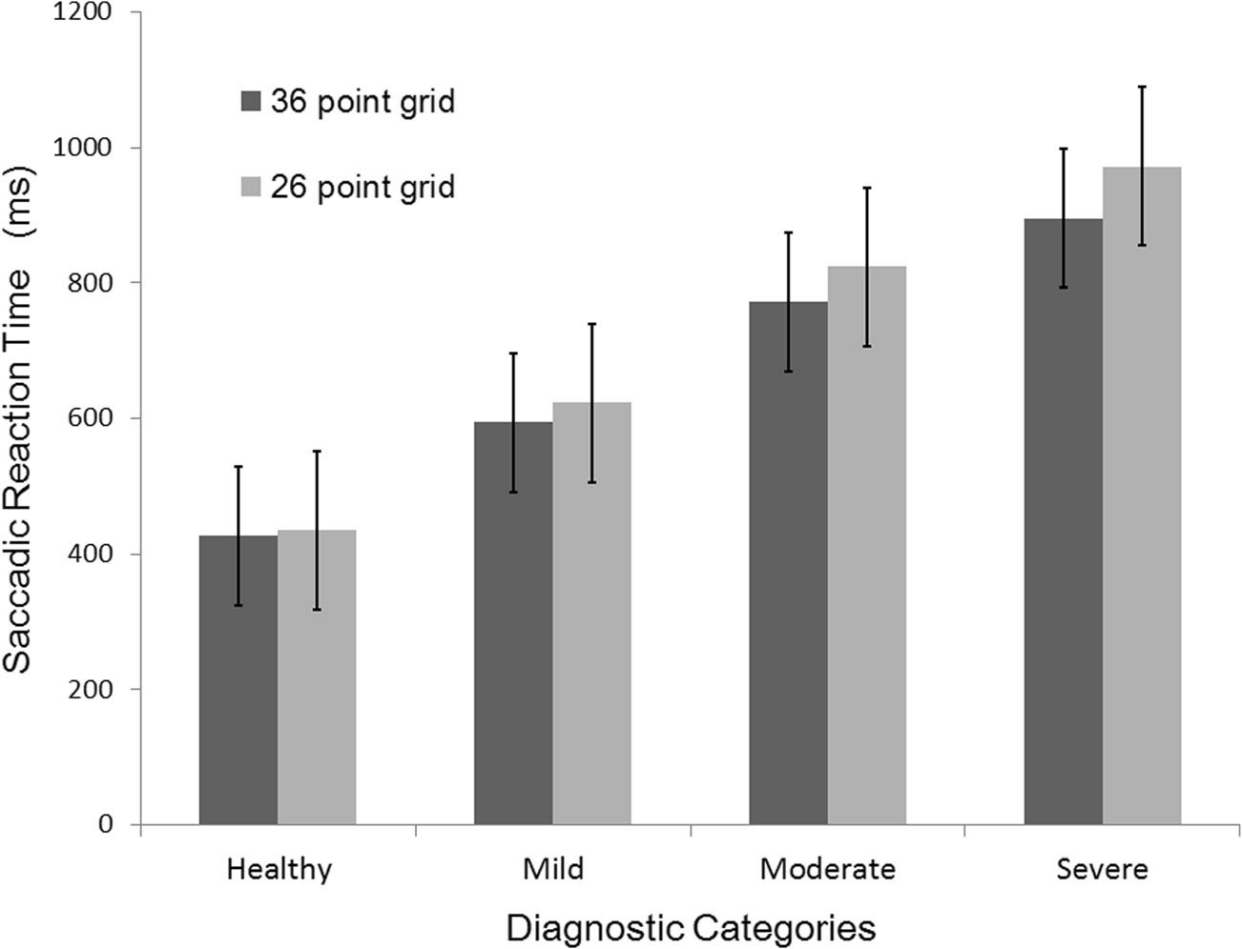
Comparison of Saccadic Reaction Time values obtained from 36 and 26 location grid

One-way ANOVA was performed to compare the SRT values within each diagnostic category obtained from both the grids. When the 36 location grid was analyzed, a significant difference in SRT values (*p* < 0.05) was noted between each of the diagnostic categories (healthy and mild, moderate and severe glaucoma). The same results were found when this analysis was carried out with the SRT values obtained from the 26 location grid (p < 0.05). The Tukey post-hoc test revealed that increasing disease severity resulted in increased SRT values. No significant difference in SRT values were noted between 36 and 26 location grids. Figure 6 illustrated the comparison of Saccadic Reaction Time values obtained from 36 and 26 location grid.

Although significant on a group level, not all glaucoma patients had a delayed reaction time. Based on a fivefold cross validation analysis, an overall classification accuracy of 90.4% (10.9) which was considered as an internal validation of the modified grid.

Finally, the average test duration (per eye) using the 36 location grid was 3.2 ± 0.3 min and 5.3 ± 0.6 min respectively among healthy subject and glaucoma patients. After the modification, a small pilot in five healthy subject and five glaucoma patients showed a test duration of 1.2 min ± 0.3 and 2.9 ± 0.6 min, respectively.

## Discussion

This study aimed at developing a screening protocol for detecting glaucomatous visual field loss by utilizing an ideal screening approach, which can be economical, easily administrable, rapid and reliable. The use of eye tracking technology offers a non-invasive, low-cost, easily administrable and objective method for measuring Saccadic Reaction Time (SRT) that can be used as an index for evaluating the functional visual aspects [7, 8].

As the intention was to screen for glaucomatous defects, maximum preference was given to evaluate the peripheral visual field areas. Therefore, the developed test grid had 26 stimulus locations comprising of four central locations and 22 test locations in the peripheral zones, predominantly testing nasal, superior and inferior of the visual field. The four central test locations were placed at slightly eccentric positions, i.e. 8 degrees from the grid center. This might trigger the false categorization of glaucomatous defects involving only central visual field as healthy ones, but this proportion might be insignificant as the early glaucomatous defects tend to affect Bjerrum area [16, 17]. Two points at the blind spot region were eliminated due to the high variability of reaction times reported in the literature [13, 18].

The evaluation of the remaining 36 point grid was carried out among healthy and glaucoma subjects who were sub-classified into mild, moderate and severe stages of glaucoma. The study sample intentionally had a high proportion of mild followed by moderate cases with relatively few severe cases. Inclusion of a large proportion of severe defects of glaucoma would have resulted in the elimination of valuable test locations, since in this group the number of unseen points due to visual field defects is higher. Using this approach we optimized the screening grid for detecting early glaucomatous defects.

To improve the reliability of assessing saccades, certain specific features were integrated into the test protocol. A peripheral stimulus was projected only when central fixation was stable. In addition, an overlap paradigm was used, meaning that the central fixation stimulus was kept visible even during the projection of subsequent peripheral stimuli, which is a similar approach used in HFA. When a central stimulus disappears, it might trigger search behavior. Despite this, a significant difference in eye movement responses was noted between healthy subjects and glaucoma patients (Table 2). In spite of instructions to not search for peripheral stimuli, glaucoma patients still exhibited more searching behavior than healthy ones, which is in agreement with previous studies [8]. This behavior might reflect the visual adaptation that glaucoma patients develop as a compensatory action for their field defects. Observation of these eye movement patterns may give insight into the real life impact of glaucomatous optic neuropathy. The percentage of false negative responses was expected to be minimal as EMP depended on the natural eye movement reflex response. The current algorithm could not incorporate an index for monitoring the possible false negative responses as we felt it would to lengthen the test duration. The test administration was noted to be examiner friendly, as the need for continuous monitoring of the subject’s response and repeated instructions were minimal.

Since perimetry is a psychophysical procedure that shows intra-test and inter-test fluctuations and variability, the Receiver Operating characteristic (ROC) curve was considered to be an efficient approach in identifying the test locations for developing the screening grid. The Area Under the Curve (AUC) summarizes the entire location of the ROC curve instead of considering single measures of sensitivity and specificity [21]. Therefore, AUC values were considered as an index of accuracy. We interpreted the AUC of a particular stimulus location as the probability to have high risk of glaucomatous damage. From Fig. 6 and supplementary table 1 it is apparent that this strategy did result in the elimination of locations, which are suspected to have poor discriminating value from clinical evidence. Faster reaction times were noted for stimulus projected at 276 cd/m^2^ in comparison with 214 cd/m^2^ and this observation was in accordance with the previous literature [13]. Mean SRT values obtained from 36 and 26 location grids were compared across four diagnostic categories, i.e. healthy, mild, moderate and severe glaucoma (Fig. 6). The difference in mean SRT (obtained from two grids) ranged from 7.5–76.8 ms, which was not statistically significant, i.e. the mean SRT values did not show a significant change even after the removal of ten locations.

Even though Pel et al. measured the SRT values using comparable background and stimulus intensity levels we used in our setup, our healthy subjects exhibited higher SRT values [11]. A possible explanation could be the higher mean age of our study sample. A similar approach was used in the work published by Thepass et al. on the effect of cataract on EMP. Here, higher luminance intensity levels were used, up to ~450 cd/m^2^, which presumably explains the faster SRT values they reported compared to our results [20].

A five fold cross validation analysis was done on the global SRT values of all subjects to have a first evaluation of the classification ability of the 26 location grid. In this method the original data sample (*n* = 104) was randomly partitioned into five subsamples, and one was left out in each iteration. This method was adapted because repeated random sub-sampling results in eventually using all subsets as both training and validation data whereas they are considered exactly once for validation. In this study, a single mean global SRT value per subject, as calculated from the 26 locations, was used for calculating the accuracy which is a rough estimate of the tested visual field responsiveness. A refinement might be achieved when a point-wise analysis is done to further evaluate its screening ability in identifying healthy subjects and patients with different grades of glaucoma. A clinical comparison of EMP screening grid should be done preferably with the current standard method (Frequency Doubling Perimetry) and the test-retest variability has to be analyzed [4, 22]. Further testing in patients diagnosed with non-glaucomatous defects would also be required.

A 90.4% of average classification accuracy was calculated while using global SRT obtained using 26 test location grid for categorizing healthy subjects and glaucoma. This suggests that SRT values can be promising index for detection of glaucomatous visual field defects. The simplicity of testing technique and strategy could potentially reduce the need for close supervision thereby allowing an easier administration of the test without relying on trained personnel. This can be a reliable approach for static perimetry specifically in pediatric and geriatric subjects.

## Conclusion

The current study resulted in an interactive EMP test grid with 26 locations which were predominantly placed in the nasal, superior and inferior areas of the visual field. Reduction of tested points resulted in reduction of test times with no significant changes in mean SRT values compared to a larger grid. Fivefold cross validation technique revealed the ability of the grid to accurately classify 90.4% of subjects into healthy and glaucoma on the basis of mean global SRT values.

## Electronic supplementary material

Supplementary Figure 1 (GIF 169 kb)

High resolution image (TIFF 950 kb)

Supplementary Table 1 (PDF 467 kb)
