# Supplementary figures and images for: Development of a test grid using Eye Movement Perimetry for screening glaucomatous visual field defects
Source: Graefes Arch Clin Exp Ophthalmol. 2017 Dec 28;256(2):371–9. doi: 10.1007/s00417-017-3872-x (PMC5790865; doi:10.1007/s00417-017-3872-x)

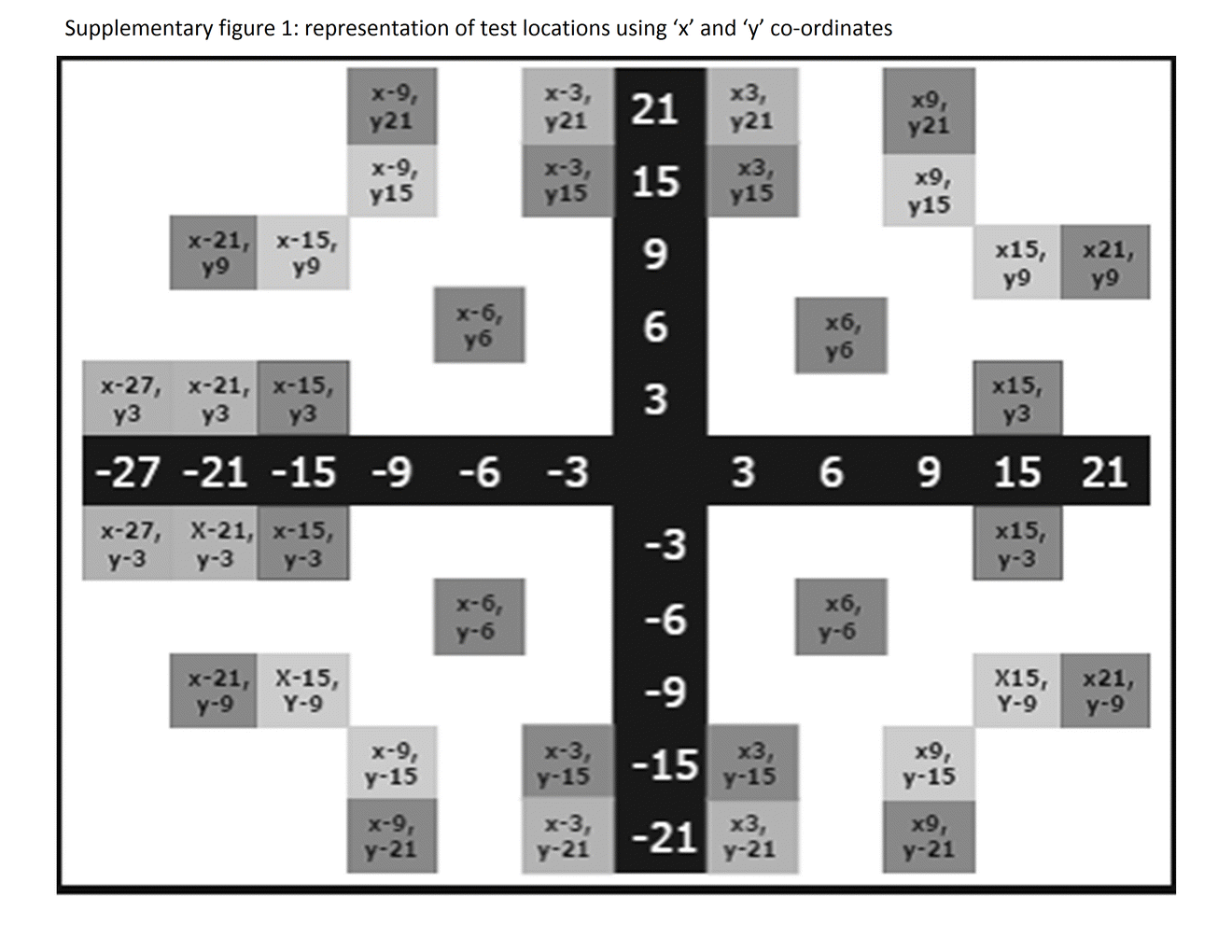

Supplement: Supplementary file 1 — (GIF 169 kb) [file 417_2017_3872_Fig7_ESM.gif]

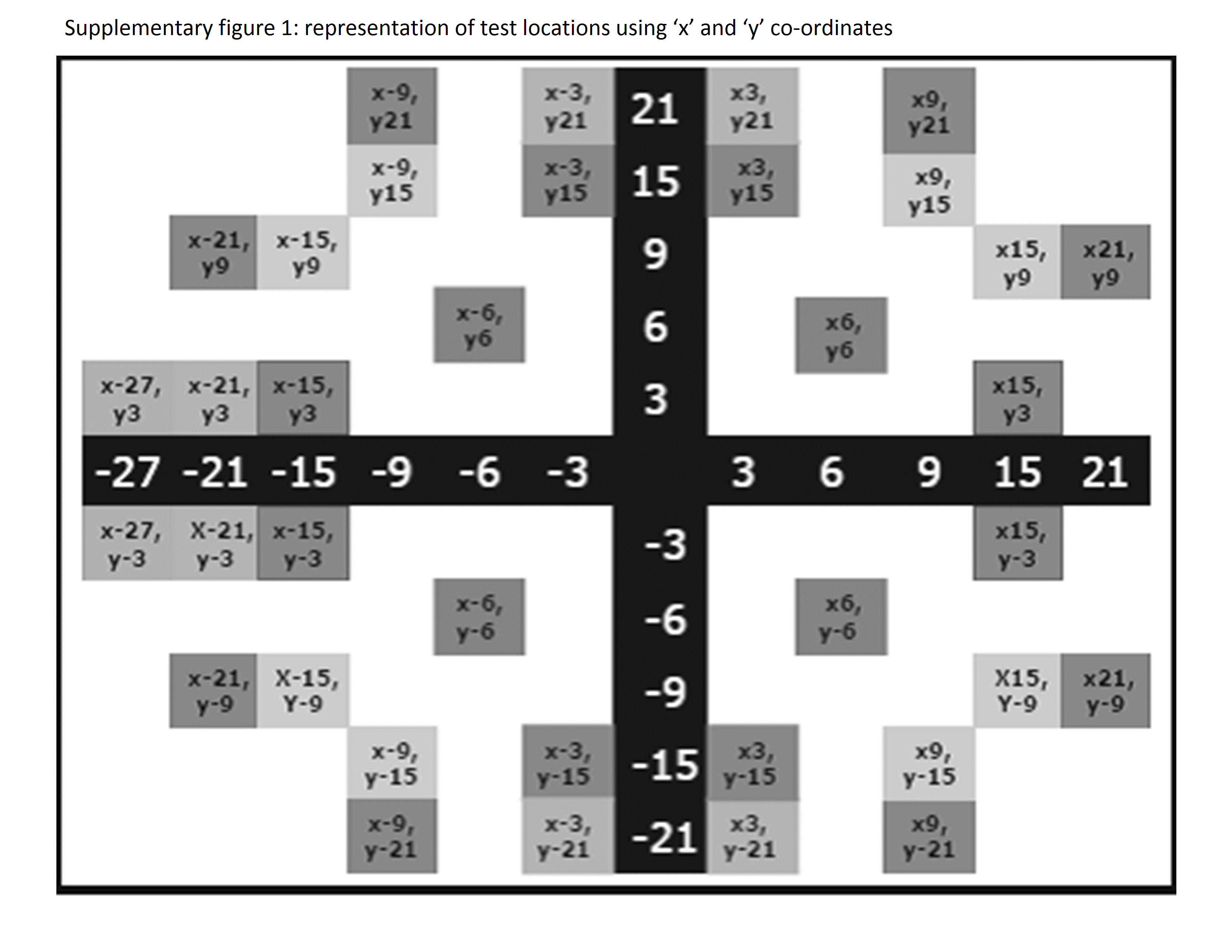

Supplement: Supplementary file 2 — High resolution image (TIFF 950 kb) [file 417_2017_3872_MOESM1_ESM.tif]
