# Supplementary material for: Development of a test grid using Eye Movement Perimetry for screening glaucomatous visual field defects
Source: Graefes Arch Clin Exp Ophthalmol. 2017 Dec 28;256(2):371–9. doi: 10.1007/s00417-017-3872-x (PMC5790865; doi:10.1007/s00417-017-3872-x)
Supplement: Supplementary file 3 — (PDF 467 kb) [file 417_2017_3872_MOESM2_ESM.pdf]

Supplementary Table 1: List of location wise AUC Cut off value, sensitivity and specificity

AUC values for four central locations							
**Tested locations**	**Area Under Curve**	**95 % CI**		**Cut off value**	**P value**	**Sensitivity (%)**	**Specificity (%)**
		**Lower**	**Upper**				
**x6,y6**	0.730	0.66	0.81	0.70	<0.001	0.70	0.71
**x6,y-6**	0.720	0.65	0.80	0.70	<0.001	0.69	0.71
**x-6,y-6**	0.710	0.62	0.78	0.65	<0.001	0.62	0.77
**x-6,y6**	0.810	0.76	0.88	0.70	<0.001	0.74	0.81
AUC values for peripheral locations							
**Tested locations**	**Area Under Curve**	**95 % CI**		**Cut off value**	**P value**	**Sensitivity (%)**	**Specificity (%)**
		**Lower**	**Upper**				
**x3,y15**	0.750	0.68	0.80	0.65	<0.001	0.60	0.69
**x3,y-15**	0.751	0.66	0.79	0.70	<0.001	0.66	0.70
**x-3,y-15**	0.770	0.73	0.85	0.70	<0.001	0.66	0.71
**x-3,y15**	0.780	0.72	0.83	0.70	<0.001	0.65	0.75
**x15,y3** [e]	0.678	0.60	0.76	0.70	<0.001	0.69	0.71
**x15,y-3** [e]	0.519	0.43	0.61	0.60	<0.001	0.60	0.69
**x-15,y-3**	0.787	0.72	0.86	0.70	<0.001	0.71	0.75
**x-15,y3**	0.796	0.72	0.87	0.70	<0.001	0.71	0.75
**x9,y15**	0.754	0.66	0.80	0.70	<0.001	0.65	0.71
**x9,y-15**	0.806	0.76	0.86	0.70	<0.001	0.68	0.75
**x-9,y-15**	0.755	0.69	0.82	0.70	<0.001	0.67	0.69
**x-9,y15**	0.798	0.71	0.86	0.70	<0.001	0.68	0.72
**x15,y9** [e]	0.792	0.73	0.86	0.80	<0.001	0.76	0.74

**x15,y-9** [e]	0.734	0.66	0.82	0.70	<0.001	0.68	0.73
**x-15,y-9**	0.759	0.70	0.82	0.70	<0.001	0.69	0.70
**x-15,y9**	0.807	0.74	0.87	0.70	<0.001	0.70	0.78
**x3,y21**	0.756	0.68	0.81	0.80	<0.001	0.76	0.74
**x3,y-21**	0.755	0.67	0.80	0.70	<0.001	0.66	0.71
**x-3,y-21**	0.756	0.70	0.82	0.80	<0.001	0.66	0.67
**x-3,y21**	0.788	0.73	0.85	0.80	<0.001	0.69	0.80
**x-21,y3**	0.763	0.70	0.83	0.70	<0.001	0.66	0.74
**x-21,y-3**	0.784	0.73	0.84	0.70	<0.001	0.68	0.76
**x9,y21** [e]	0.714	0.65	0.78	0.65	<0.001	0.66	0.65
**x9,y-21** [e]	0.770	0.71	0.83	0.70	<0.001	0.69	0.73
**x-9,y-21** [e]	0.698	0.63	0.77	0.65	<0.001	0.75	0.62
**x-9,y21** [e]	0.768	0.73	0.85	0.70	<0.001	0.70	0.77
**x21,y9** [e]	0.715	0.65	0.78	0.65	<0.001	0.67	0.65
**x21,y-9** [e]	0.738	0.67	0.80	0.65	<0.001	0.66	0.71
**x-21,y-9**	0.767	0.71	0.83	0.80	<0.001	0.68	0.78
**x-21,y9**	0.774	0.71	0.84	0.70	<0.001	0.65	0.77
**x-27,y3**	0.751	0.69	0.81	0.70	<0.001	0.70	0.78
**x-27,y-3**	0.789	0.73	0.85	0.70	<0.001	0.69	0.80

CI: Confidence Interval
[e] Eliminated locations
